# A rare case of isolated wound implantation of colorectal adenocarcinoma complicating an incisional hernia: case report and review of the literature

**DOI:** 10.1186/1477-7819-6-5

**Published:** 2008-01-17

**Authors:** Aninda Chandra, Lester Lee, Fahad Hossain, Harnaik Johal

**Affiliations:** 1 Department of General Surgery, Queen Mary's Hospital Sidcup, Sidcup, UK

## Abstract

**Background:**

The reported case illustrates an instance of colonic adenocarcinoma presenting as an isolated tumour 3 1/2 years after open surgery. The presentation was in some respects unique as it was complicated by an incisional hernia and occurred in the anterior abdominal wall. A literature review was performed.

**Case presentation:**

An 83 year old lady initially underwent an extended right open hemicolectomy for a mid-transverse colonic adenocarcinoma (T4N2M0). No adjacent structures were involved. After adjuvant chemotherapy, she was kept under regular surveillance. A CT scan and colonoscopy at one year were normal. At 18 months investigations including an ultrasound scan of the liver and a radioisotope bone scan were all negative. Over three and half years later the patient presented with an incisional hernia. Repeat CT scan and tumour markers were reported as negative. At operation, a mass was found within the anterior abdominal wall complicating the incisional hernia. This mass was widely resected and a laparotomy performed. Histology confirmed an adenocarcinoma of colonic origin extending to one of the lateral margins. A post-operative PET scan confirmed the absence of intra-abdominal pathology.

**Conclusion:**

The literature regarding recurrence of colonic tumours after open surgery reports low incidences of this occurring within abdominal incisions. The literature indicates prognosis is poor, but the numbers are small and distinction is often not made between isolated recurrence and those with other sites of tumour recurrence. In order to avoid missing isolated wound implantation, careful consideration should be given to those who present with new pathology related to previous cancer surgery incisions, both clinically and radiologically.

## Background

The prognosis associated with colorectal cancer has significantly improved due to advances in early diagnosis and therapeutic techniques. The post-operative follow-up of such patients remain an integral part of management due to the potential for recurrent disease. The prevalence of loco-regional recurrence or metastatic disease, especially to the liver and lung, is well recognised and hence forms the main focus of follow-up imaging investigation.

The question of wound recurrences after laparotomy has been infrequently addressed in the literature [1,2], in contrast to port-site recurrences. This was due to a high incidence of early port-site/wound recurrences being reported after laparoscopic resection of colorectal malignancy [2,3]. Prospective randomised trials [4,5] showed however no difference between open and laparoscopic groups with less than a 1% wound recurrence rate, with at least a four year follow-up. Isolated wound recurrences of colorectal adenocarcinoma presenting after open surgery is rare: the literature reports an incidence of 0% to 0.4% of all resections when followed prospectively [6-8]. Isolated port-site recurrence after laparoscopic resection in large trials is also rare [4,5,8-10]; with one group [10] reporting an incidence of 0.2%.

CT imaging is an effective modality in diagnosing recurrences; however it may be limited in cases where isolated wound recurrences following open surgery co-exist with other benign pathologies. The case report relates to a patient presenting with an anterior abdominal wall hernia 3 1/2 years after open surgery, who was found to have an incidental anterior abdominal wound tumour at operation, despite a pre-operative CT scan reported as normal.

## Case presentation

An 83 year old lady initially underwent via a midline vertical incision, an extended right hemicolectomy in 2003. She had presented with weight loss with no previous medical or surgical history. Functionally she was independent and self-caring. Pre-operative radiology (including a staging CT scan) showed a mid-transverse colonic lesion. Colonoscopy revealed no other intra-colonic lesions and tumour markers were normal.

At operation, there was no invasion into other structures or the anterior abdominal wall. Histology demonstrated a T4 N2 Mx adenocarcinoma in the transverse colon. The serosa had been breached but the tumour had been completely excised. The apical node was clear but 4 out of 11 nodes were involved. The case was discussed pre- and post-operatively in the Gastro-intestinal (GI) multi-disciplinary meeting (MDM) and staged as T4 N2 M0 (Dukes C1). Adjuvant chemotherapy was offered to the patient, who subsequently underwent a weekly course of bolus 5FU & Folinic acid. This was well tolerated with only grade I nausea and mild hair loss and was completed at six months post-operation.

The patient was seen regularly in clinic on a three monthly basis. At one year, the surveillance CT scan (chest, abdomen and pelvis) was unremarkable as was colonoscopy. At 18 months, the patient complained of lower back pain in April 2005. In view of her history a chest X-ray, tumour markers and ultrasound scan of the liver were ordered. These were all negative. A radioisotope bone scan was performed. The scan showed only lumbro-sacral arthritis and her pain resolved with simple analgesia.

At three and a half years post-surgery, she reported some mild abdominal discomfort and distension. She attributed this to her incisional hernias, at the site of the midline scar. These had progressively worsened in size as had her symptoms. On examination, she was found to have two incisional hernias which lay 20 mm above and 20 mm below her umbilicus and were 30 mm and 40 mm respectively in diameter. A contrast enhanced staging CT of the chest, abdomen and pelvis was performed. A midline ventral hernia was noted on transverse slices of the CT image but no focal lesion was reported. The anastomotic site appeared normal with no recurrent growth or lymphadenopathy otherwise seen. Tumour markers were not elevated (CEA = 3, CA 19-9 = 3, CA125 = 5). An incisional hernia repair was subsequently arranged and a specialised mesh was ordered. The provisional plan was to place the mesh behind the anterior abdominal wall (anterior to the peritoneum). As there were two large defects which were closely related, a 20 cm × 15 cm Bard Composix-Mesh^® ^(C. R. Bard, Inc., 730 Central Aves Murray Hill, New Jersey, 07974, USA) was ordered

**Figure 1:**
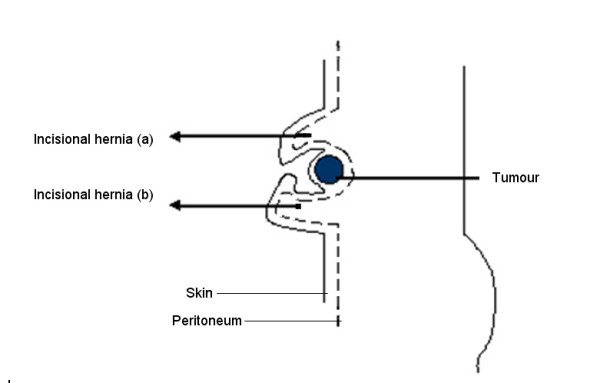
Sagital schematic view of tumour recurrence in anterior abdominal wound complicated by two incisional hernias: A – incisional hernia 20 mm above umbilicus (30 mm diameter). B – incisional hernia 20 mm below umbilicus (40 mm diameter).

At operation in 2007, a further midline incision was performed. Following division of skin and subcutaneous tissue the anterior abdominal wall was visualised. The two incisional hernia sacs were each identified and freed from their attachments to the anterior abdominal wall allowing pre-peritoneal access. At this point it became apparent, that the tissue in between the two incisional hernias was not dense scar tissue. On palpation a hard mass measuring 20 mm × 20 mm in diameter was found situated within the anterior abdominal wall. This was not attached to peritoneum. Thus it appeared as if it may be an isolated recurrence (Figure 1). The mass was excised with a wide margin and sent for histology. A formal laparotomy was performed and no intra-abdominal recurrence or peritoneal seedlings were noted.

As defect following the wide excision was closed using the Bard Composix-Mesh^®^. This was attached with 3/0 Prolene to parietal peritoneum using continuous sutures as a modified sub-lay technique. The rectus sheath was approximated but not apposed with 1/0 nylon to allow a tension free repair. A vacuum drain was placed superficial to the anterior rectus sheath. Closure was with interrupted subcutaneous 3/0 Vicryl sutures and clips to skin. The post-operative course was uncomplicated.

**Figure 2:**
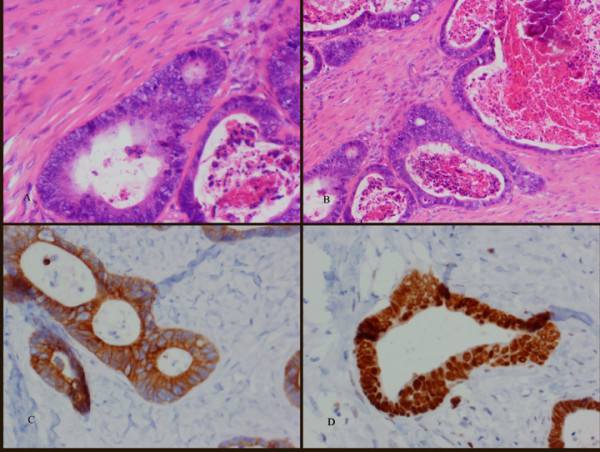
**A and B) **Photomicrograph showing malignant glands typical of adenocarcinoma lined by atypical cells with hyperchromatic nuclei. There is an increase in mitotic activity within the cells and the presence of necrotic material. Stained with haematoxylin & eosin. **C) **Immunohistochemistry with CK20 showing tumour cell cytoplasm stained. **D) **Immunohistochemistry with CDX2 staining showing prominent nuclei of tumour cells. CK20 and CDX2 are consistent with cells of colorectal origin. **Note: **Original magnifications **a **– **d **20×.

The mass which measured 40 mm × 40 mm × 30 mm. Histologically, it consisted of fibro-connective tissue infiltrated by a moderately differentiated adenocarcinoma. The tumour cells were seen to involve one of the lateral surgical margins. There was no superior or inferior extension of the tumour. Subsequent immuno-histochemistry was positive for CK20 and CDX2 and negative for CK7 (Figure 2). This was characteristic of tumour cells arising from a colorectal origin and in keeping with the original pathology.

**Figure 3:**
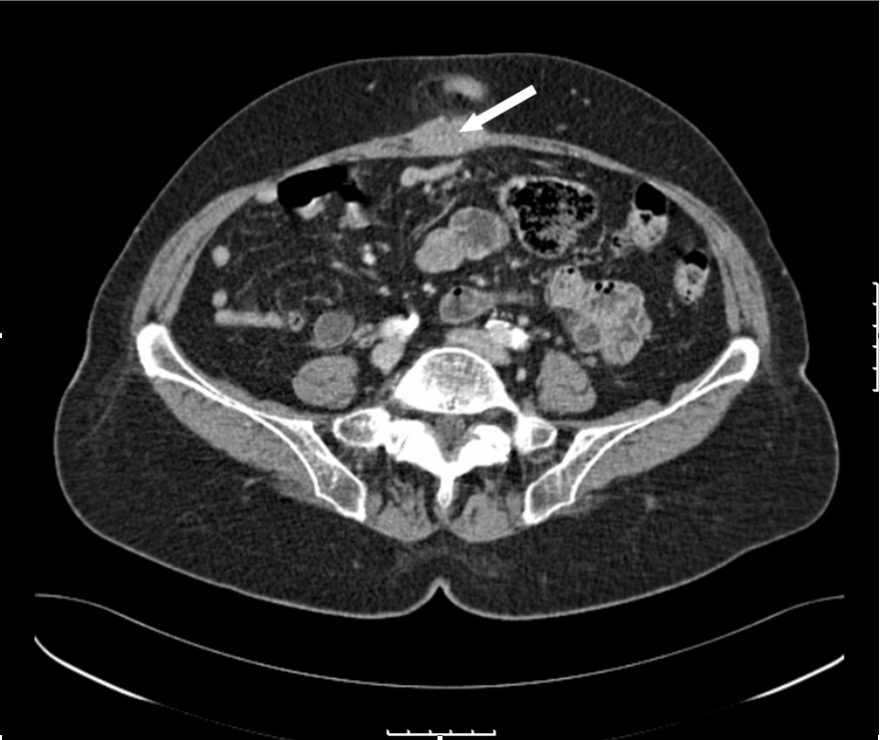
CT scan of abdomen showing soft tissue mass in the anterior abdominal wall (white arrow). The ventral incisional hernia is seen on this slice and was arising cranially but lies superiorly to the mass.

The case was discussed again in the GI MDM. On review of the scans, a 3.6 × 1.6 cm nodule was seen in the midline on the anterior abdominal wall just inferior to the hernia (Figure 3). The absence of intra-abdominal recurrence was reconfirmed, postoperatively with a repeat PET scan. The patient was subsequently seen in outpatients' clinic and the possible management strategies were outlined in the presence of the colorectal specialist nurse and the patient's surgical consultant.

The presentation and case above was novel to the department. As such an extensive literature search was performed using EMBASE and MEDLINE to find similar cases and related articles. The prognosis obtained from the literature following surgery to attempt clearance was not significantly better then adjuvant therapy. In view of this and the potential complications, she requested to be referred to an oncologist for consideration of palliative chemo-radiotherapy.

## Discussion

After open surgery, tumour recurring within a surgical wound is uncommon but probably underestimated [7]. Two large prospective trials which looked at recurrence of colonic tumours after open surgery reported low incidences of abdominal scar recurrence; Hughes et al [6] reported a figure of 11 out of 1603 patients (0.7%) while Reilly et al [7] documented 9 cases from 1711 patients (0.5%). Isolated wound recurrence is an even rarer phenomena with laparotomy or radiology often demonstrating tumour recurrence at other sites [6,7,11]. Isolated occurrence occurred in the study by Reilly et al [7] in only 3 patients with abdominal or perineal wound recurrences (0.2%). Hughes et al [6] stated that isolated recurrences were found in only 6 abdominal scar cases (0.4%). As the study was from 1950 to 1980, this predates CT scan usage, therefore the actual incidence of isolated recurrence would probably have been lower if current imaging modalities had been applied.

In comparison to open surgery, wound recurrences at port sites after laparoscopic surgery [12,13] were initially thought to be more common [7]. Subsequently more objective prospective randomised trials [13,14] have showed no significant difference in recurrence compared to open surgery. Two large studies [4,5] showed less than 1% wound recurrence in both laparoscopic resections and open colectomies, with a median follow-up of at least 4 years. Hartley *et al*., [8] found that all wound recurrences in their prospective study, comparing laparoscopy and open resection, were associated with advanced intra-peritoneal disease. Isolated port-site recurrence after laparoscopic resection in large trials is rare [4,5,8-10]; Silecchia *et al*., [10] reported an incidence of 0.2% when cases were followed prospectively.

Isolated tumour occurring at a point distal arises from a combination of different factors. An important factor is considered to be residual viable tumour cells left in the abdomen. These can be cells exfoliated from the tumour [15] or by contamination of surgical equipment used intra-operatively [16]. These cells can then disseminate to the site of recurrence or spread may occur by direct iatrogenic implantation. The presence of tumour cells at a site does not necessitate implantation and other local factors need to be involved [17].

The trauma of surgery results in an inflammatory response which has been shown to enhance the successful implantation of exfoliated tumour cells in animal models [18]. Inflammatory cytokines such as TNF-α, IL-1 and IL-6 are involved in angiogenesis, which is fundamental step in tumour development. These inflammatory cytokines together with VEGF can be found in surgical wounds. They can also increase the expression of adhesion molecules and the adhesion of tumour cells becomes more successful after the infliction of surgical trauma [17]. The environment of a healing incision can therefore not only assist in the development of tumour cells, but also to their adhesion to cell surfaces. Wound implantation therefore may be more likely in the early post operative period during healing. The relatively late presentation of tumour recurrence 3 1/2 years after initial surgery [1,4] as described in the case report was an additional confounding factor in the tumour not being detected pre-operatively.

There were a number of clinical issues arising from this case. Although disease recurrence had been the indication for performing the preoperative investigations, the relatively rare occurrence of an isolated tumour within the surgical wound (in the absence of intra-abdominal disease or chest metastasis) was not appreciated by the consultant radiologist when reporting on the CT scan. The complexity of the incisional hernia with its components lying above and below the tumour also contributed to the difficulty in picking up the lesion (Figure 1). This was compounded by normal tumour markers which included a normal CEA result. The identification of the tumour was complicated by the presence of the incisional hernia. In the majority of reported cases in the literature (>90%), recurrence was manifested within 2 years of surgery [1,4] where as in the case reported it presented after 3 1/2 years. In light of the intra-operative findings, the case and the CT scan were presented at a joint radiological/surgical/oncological meeting. The lesion was retrospectively identified on the pre-operative CT images (Figure 3). This finding if it had been noted pre-operatively would have altered management especially with regards to pre-operative chemo-radiotherapy and the surgical approach.

In the case report, there was no clinical evidence of tumour within the wound pre-operatively. A combined PET/CT scan was found by Goshen et al [11] to be extremely sensitive in detecting abdominal wound recurrences in patients with advanced disease as small as 1 cm in diameter. However if this were to be used routinely as an imaging modality to exclude recurrence, it would be expensive.

Given the involvement of the surgical margins, the options available were either radical re-excision or radiotherapy. Hughes et al [6] described a 5 year survival of 0% and Reilly et al [7] of 27% in their surgical incisional recurrences. The former study based from 1950 to 1980 may have not benefited from the advances in adjuvant chemotherapy in the last few decades. Reilly et al [7] could not detect a significant difference in survival (or of time to recurrence) between the group with isolated recurrence versus those with other sites of involvement, although the numbers were noted to be small. Based on the literature the prognosis was deemed as poor even with resection. Excision and current adjuvant chemo-radiotherapy may improve outcome but there is little definitively published.

## Conclusion

The case reported illustrates an instance of colonic adenocarcinoma recurring as an isolated tumour after open surgery. Its presentation was unique as it was complicated by an incisional hernia and presented in the anterior abdominal wall. Tumour markers were negative and there was no intra-abdominal pathology. Wound implantation in an incisional scar after open surgery is rare, particularly when it is isolated and presentation is more than two years after the original surgery.

The literature indicates prognosis is poor, but the numbers are small and distinction is often not made between isolated incisional wound implantation and those with other sites of tumour recurrence or co-existent intra-abdominal malignancy. Further studies on this would shape current practice.

There were a number of factors which arose in this case including the CT scan report, which may have been altered by a higher index of suspicion. In order to avoid missing isolated wound implantation, careful consideration should be given to those who present with new pathology related to previous cancer surgery incisions, both clinically and radiologically.

## Abbreviations

CEA: Carcinoembryonic Antigen; CT: Computerized Tomography; GI: Gastro-intestinal; MDM: Multi-Disciplinary Meeting; PET: Positron Emission Tomography.

## Competing interests

The author(s) declare that they have no competing interests.

## Authors' contributions

Each author performed an independent literature search. AC, and LL operated upon the patient initially, critically appraised the literature and conceived the case report; HJ reviewed the literature and revised the final manuscript; FH reviewed the literature and helped in drafting the manuscript. All authors read and approved the final manuscript.

## References

[B1] Reymond MA, Bonjer HJ, Kockerling F (2000). Port-Site and Wound Recurrences in Cancer Surgery: Incidence, Pathogenesis Springer.

[B2] Schaeff B, Paolucci V, Thomopoulos J (1998). Port site recurrences after laparoscopic surgery. A review. Dig Surg.

[B3] Jacquet P, Averbach AM, Jacquet N (1995). Abdominal wall metastasis and peritoneal carcinomatosis after laparoscopic-assisted colectomy for colon cancer. Eur J Surg Oncol.

[B4] Lacy AM, Garcia-Valdecasas JC, Delgado S, Castells A, Taura P, Pique JM, Visa J (2002). Laparoscopy-assisted colectomy versus open colectomy for treatment of non-metastatic colon cancer: a randomised trial. Lancet.

[B5] The Clinical Outcomes of Surgical Therapy Study Group (2004). A comparison of laparoscopically assisted and open colectomy for colon cancer. N Engl J Med.

[B6] Hughes ES, McDermott FT, Polglase AL, Johnson WR (1983). Tumor recurrence in the abdominal wall scar tissue after large-bowel cancer surgery. Dis Colon Rectum.

[B7] Reilly WT, Nelson H, Schroeder G, Wieand HS, Bolton J, O'Connell MJ (1996). Wound recurrence following conventional treatment of colorectal cancer. A rare but perhaps underestimated problem. Dis Colon Rectum.

[B8] Hartley JE, Mehigan BJ, MacDonald AW, Lee PW, Monson JR (2000). Patterns of recurrence and survival after laparoscopic and conventional resections for colorectal. Ann Surg.

[B9] Mehta PP, Griffin J, Ganta S, Rangraj M, Steichen F (2005). Laparoscopic-assisted colon resections: long-term results and survival. JSLS.

[B10] Silecchia G, Perrotta N, Giraudo G, Salval M, Parini U, Feliciotti F, Lezoche E, Morino M, Melotti G, Carlini M, Rosato P, Basso N, For the Italian Registry of Laparoscopic Colorectal Surgery (2002). Abdominal wall recurrences after colorectal resection for cancer: results of the Italian registry of laparoscopic colorectal surgery. Dis Colon Rectum.

[B11] Goshen E, Davidson T, Aderka D, Zwas ST (2006). PET/CT detects abdominal wall and port site metastases of colorectal carcinoma. Br J Radiol.

[B12] Berends FJ, Kazemier G, Bonjer HJ, Lange JF (1994). Subcutaneous metastases after laparoscopic colectomy. Lancet.

[B13] Lacy AM, Delgado S, Garcia-Valdecasas JC, Castells A, Pique JM, Grande L, Fuster J, Targarona EM, Pera M, Visa J (1998). Port site metastases and recurrence after laparoscopic colectomy. A randomized trial. Surg Endosc.

[B14] Basha G, Penninckx F, Mebis J, Filez L, Geboes K, Yap P (1999). Local and systemic effects of intraoperative whole-colon washout with 5 per cent povidone-iodine. Br J Surg.

[B15] Umpleby HC, Fermor B, Symes MO, Williamson RC (1984). Viability of exfoliated colorectal carcinoma cells. Br J Surg.

[B16] Alagaratnam TT, Ong GB (1977). Wound implantation – A surgical hazard. Br J Surg.

[B17] Oosterling SJ, van der Bij GJ, van EM, van dS (2005). Surgical trauma and peritoneal recurrence of colorectal carcinoma. Eur J Surg Oncol.

[B18] Raa ST, Oosterling SJ, van der Kaaij NP, van den Tol MP, Beelen RH, Meijer S, van Eijck CH, van der Sijp JR, van Egmond M, Jeekel J (2005). Surgery promotes implantation of disseminated tumor cells, but does not increase growth of tumor cell clusters. J Surg Oncol.

